# Editorial: Advancement in malaria treatment

**DOI:** 10.3389/fcimb.2025.1628923

**Published:** 2025-05-28

**Authors:** Sarika Gunjan, Manish Goyal

**Affiliations:** ^1^ Department of Pathology, Medical College of Wisconsin, Milwaukee, WI, United States; ^2^ Department of Molecular and Cell Biology, Boston University Goldman School of Dental Medicine, Boston, MA, United States

**Keywords:** malaria, plasmodium, antimalarial drug resistance, artemisinin-based combination therapies (ACTs), PfCoronin, spleen-dependent protein 1, cerebral malaria

Throughout human history, infectious diseases have posed a serious threat to humanity, particularly through the prevalence of devastating parasitic diseases. Apicomplexan parasites play a significant role in this landscape, impacting populations worldwide and leading to diseases such as toxoplasmosis, cryptosporidiosis, babesiosis, and malaria (Kaminsky and Maser, 2025). Malaria, caused by the Plasmodium parasite, is especially concerning due to its life-threatening nature. While initial symptoms of malaria often manifest as fever, chills, headaches, and muscle aches, severe cases can escalate to critical complications such as cerebral malaria and multiorgan failure (Milner, 2018). This parasitic disease poses a significant health crisis in tropical and subtropical regions, particularly in developing nations. According to WHO report, malaria causes roughly 263 million cases and 597,000 deaths each year across 83 countries. The African region experienced the greatest impact, with 246 million cases (94% of the global total) and 569,000 deaths (95% of the global total), and, tragically, young children under 5 accounted for about 76% of the fatalities (Venkatesan, 2025).

Despite centuries of effective malaria treatments, the disease tragically continues to claim hundreds of thousands of lives annually. The growing threat of drug-resistant malaria parasites severely limits the effectiveness of current frontline chemotherapies in real-world settings (Haldar et al., 2018; van der Pluijm et al., 2021). This necessitates urgent and innovative research to discover new drugs capable of overcoming the rapid evolution of resistance. The current Research Topic focuses on the latest advancements in antimalarial drug targets, antimalarial resistance, innovative therapeutic approaches, and preventative measures. Our main goal for this Research Topic is to deepen our understanding of drug resistance mechanisms and foster the development of new prophylactic and therapeutic agents and the repurposing of existing drugs. This Research Topic features three original research articles and three review articles, summarized as follows.

Artemisinin-based combination therapies (ACTs), the primary defense against malaria, are increasingly threatened by drug resistance (Haldar et al., 2018; van der Pluijm et al., 2021; White and Chotivanich, 2024). To maintain their effectiveness, particularly in high-endemic drug resistance areas, routine surveillance of parasite genetic variation is very crucial. The first article in this Research Topic focuses on tracking these genetic variations in the malaria parasite within Lagos, Nigeria (Ajibaye et al.). It investigates mutations in the *Plasmodium falciparum* actin-binding protein (Pfcoronin) that are associated with decreased sensitivity to artemisinin (ART). The authors shed light on the novel Pfcoronin mutations that may lead to persistent malaria infections following ACT treatment (Ajibaye et al.). The identified mutations could have significant implications for parasite clearance and may inform future research on the evolving tolerance to ART in Nigeria and other West African nations.

Employing a related strategy in the second article, the authors have utilized bibliometrics and knowledge mapping tools to analyze the research landscape of antimalarial drug resistance. This method involves applying mathematical and statistical tools to visualize research databases. Drawing upon 2559 relevant articles published between 2015 and 2023 from the Web of Science Core Collection (WOSCC), the authors represent the first visual bibliometric analysis of antimalarial drug resistance (Zhang et al.). The results underscored significant research areas, including the mechanisms behind the action and resistance to artemisinin-based combination therapies (ACTs)—highlighting aspects like heme activation, targeting vital processes in the parasite, such as REDOX responses, lipid and protein synthesis, and the frequency of the Pfkelch13 R561H mutation. Additionally, the study noted the emergence of innovative antimalarial drugs, represented by DDD107498, MMV390048, DSM265, and Ozonides, all designed to overcome the shortcomings of existing first-line treatments (Zhang et al.).

Recognizing the critical need for new antimalarials, the third article centers on a new antimalarial candidate, M5717. This promising lead molecule, currently undergoing clinical trials, targets multiple stages of the malaria parasite through the inhibition of elongation factor 2 (PfeEF2) (McCarthy et al., 2021). However, its use has revealed an issue with the persistence of the parasite in the circulation after treatment. In this article, the authors shed light on the pharmacological effect of the M5717 on *P. falciparum* and explore its clearance dynamics. The study found that the delayed clearance of parasites from the bloodstream is linked to their impaired ability to effectively modify host red blood cells (RBCs) following M5717 treatment (Parkyn Schneider et al.). These findings highlight challenges associated with M5717 treatment and suggest that innovative strategies may be necessary to address this unique resistance mechanism (Parkyn Schneider et al.).

Blocking malaria transmission through its vector control also offers another promising tool for malaria control. The next article of the issue, which is a review article, focuses on vector control, particularly by integrating traditional methods with emerging Wolbachia-based interventions. In this review, the authors highlight how Wolbachia, a natural bacterium, can reduce mosquito lifespan and parasite transmission (Mushtaq et al.). The authors emphasize Wolbachia’s potential within a multi-dimensional strategy involving community and environmental efforts to improve malaria control in various countries. The paper concludes that continued research and international collaboration are crucial for the real-world application of this sustainable strategy (Mushtaq et al.).

Artemisinin-based combination therapies (ACT) lower parasite levels but do not fully resolve the harmful processes of cerebral malaria (CM)-like blood-brain barrier damage, endothelial problems, and hyperinflammation. Immunomodulatory drugs, which play a key role in combating severe malaria as an adjuvant, offer considerable potential to improve treatment outcomes and reduce mortality in CM (John et al., 2010). In this context, the next mini-review article in this Research Topic explores the potential of adjuvant therapies for CM (Bensalel and Gallego-Delgado). Overall, the authors summarized promising therapeutic targets and treatments for CM. A thorough understanding of both the advantages and potential side effects of these therapies is vital for creating effective treatment strategies that tackle the complexities of malaria and its complications (Bensalel and Gallego-Delgado).

Beyond *P. falciparum* malaria, asymptomatic chronic infection caused by *Plasmodium vivax* represents another big challenge for the malaria eradication program (Price et al., 2020). Recent studies have shown that the spleen emerges as a key player that harbors a substantial burden of parasite biomass during asymptomatic infection. However, it is largely unknown how *P. vivax* utilizes circulating EVs to communicate with the human spleen, thereby facilitating the formation of cryptic intrasplenic infections. The last article of this Research Topic investigates this by exploring the role of a novel *P. vivax* Spleen-Dependent Protein 1 (PvSDP1), whose expression and localization depend on the spleen (Ayllon-Hermida et al.). Using CRISPR/Cas9 and single-cell RNA sequencing, the authors reveal the spleen’s crucial role in storing and interacting with the parasite during chronic asymptomatic *P. vivax* infection. The authors concluded that understanding these interactions may lead to future therapies targeting the parasite within the spleen for more effective malaria control (Ayllon-Hermida et al.).

In conclusion, this Research Topic highlights the significant advancements we have made recently and shows the global dedication to developing innovative solutions for malaria eradication. It underscores the key breakthroughs in understanding drug resistance mechanisms, the development of chemotherapeutic agents, and creative strategies for repurposing existing medications. We sincerely thank everyone who shared their expertise and efforts in this important work.
